# Thyroid uptake and scintigraphy using ^99m^Tc pertechnetate: standardization in normal individuals.

**DOI:** 10.1590/S1516-31802002000200004

**Published:** 2002-03-02

**Authors:** Celso Darío Ramos, Denise Engelbrecht Zantut Wittmann, Elba Cristina Sá de Camargo Etchebehere, Marcos Antonio Tambascia, Cleide Aparecida Moreira Silva, Edwaldo Eduardo Camargo

**Keywords:** Thyroid, Scintigraphy, Uptake, [^99m^Tc] pertechnetate, Standardization, Tireóide, Cintilografia, Captação, Pertecnetato-^99m^Tc, Valores normais

## Abstract

**CONTEXT:**

Thyroid uptake and scintigraphy using ^99m^Tc-pertechnetate has proven to be more advantageous than with ^131^I-iodide, since the images have better quality, the procedure is faster and the patient is submitted to a lower radiation dose.

**OBJECTIVE:**

The purpose of this study was to standardize a simple and fast methodology for performing thyroid uptake and scintigraphy and to determine the normal values for ^99m^Tc- pertechnetate uptake.

**TYPE OF STUDY:**

Prospective, non-randomized.

**SETTING:**

Division of Nuclear Medicine, Department of Radiology, School of Medical Sciences, Campinas State University.

**PARTICIPANTS:**

The study consisted of 47 normal individuals, 30 women and 17 men, with ages ranging from 19 to 61 years (mean of 33 years).

**PROCEDURES:**

The laboratory assessment of thyroid function consisted of serum dosages of ultra-sensitive thyroxin and thyrotrophin. Twenty minutes after an intravenous injection of 10 mCi (370 MBq) of ^99m^Tc-pertechnetate, the images were obtained on a computerized scintillation camera equipped with a low-energy high-resolution parallel hole collimator.

**RESULTS:**

All the individuals were euthyroid both on clinical and laboratory evaluation. The baseline thyroid ^99m^Tc-pertechnetate uptake ranged from 0.4 to 1.7%. The uptake values obtained in these normal individuals showed that 95% presented a thyroid uptake that ranged from 0.4 to 1.5% of the injected dose.

**CONCLUSION:**

Theassessment of thyroid structure and function using ^99m^Tc-pertechnetate is a simple, fast and efficient method, which could easily become a part of the routine studies in nuclear medicine laboratories.

## RESUMO

**CONTEXTO:**

A utilização do pertecnetato-^99m^Tc para captação e cintilografia da tireóide exibe vantagens em relação ao iodeto-^131^I, como menor exposição do paciente à radiação, imagens de melhor qualidade e maior rapidez para a realização do procedimento.

**OBJETIVO:**

Padronizar uma metodologia simples e rápida para a realização de captação e cintilografia da tireóide e determinar os valores normais de captação tireoidiana de pertecnetato-^99m^Tc.

**TIPO DE ESTUDO:**

Prospectivo, não-rando-mizado.

**LOCAL:**

Serviço de Medicina Nuclear, Faculdade de Ciências Médicas da Universidade Estadual de Campinas, Campinas, Brasil.

**PARTICIPANTES:**

Estudamos 47 indivíduos normais, 30 mulheres e 17 homens, com idades variando entre 19 e 61 anos (média 33 anos).

**PROCEDIMENTOS:**

A avaliação laboratorial da função tireoidiana foi feita por meio de dosagens séricas de tiroxina e tireotrofina. As imagens foram adquiridas 20 minutos após a injeção venosa de 10 mCi (370 MBq) de pertecnetato-^99m^Tc em uma câmara de cintilação computadorizada equipada com colimador de baixa energia, alta resolução e furos paralelos.

**RESULTADOS:**

Todos os indivíduos eram clínica e laboratorialmente eutireoidianos. A captação tireoidiana basal de pertecnetato-^99m^Tc variou de 0,4 a 1,7%. A análise estatística evidenciou que 95% dos indivíduos normais estudados apresentaram valores de captação entre 0,4 a 1,5% da dose injetada.

**CONCLUSÃO:**

A avaliação da estrutura e função tireoidianas com o uso do pertecnetato-^99m^Tc é um método simples, rápido e eficiente, podendo ser facilmente incorporado à rotina dos serviços de medicina nuclear.

## INTRODUCTION

Thyroid gland function and structure can be evaluated using uptake and scintigraphy studies. ^131^I-iodide, which was introduced in the late thirties, was the first radiopharmaceutical used for measuring thyroid uptake, and for many years it was the main study agent used in the evaluation of thyroid function.^1^ Despite the fact that the sensitivity and specificity of *in vitro* tests for evaluation of thyroid function have evolved, thyroid uptake and scintigraphy still play an important role in various clinical situations, such as the detection of ectopic thyroid tissue in neck masses, functional assessment of single or multiple nodules, increasing the likelihood of detecting hyperthyroidism in difficult cases, identification of other causes of thyrotoxicosis and calculation of therapeutic doses of ^131^I-iodide.^2^

Studies with ^131^I-iodide have the serious disadvantage of high radiation doses to the gland (1–3 rad/mCi administered) caused by its long half-life and β*^-^* particle emission. Its main gamma photon has high energy (364 keV) that is inadequately collimated by most conventional scintillation cameras, and therefore poor quality images are produced. In the United States, the use of ^131^I-iodide for thyroid imaging has been prohibited and its use restricted to staging and follow-up of patients with differentiated thyroid carcinoma.^2,3^

Iodine-123 is a good substitute for iodine-131 because it has a shorter half-life (13 hours), a gamma photon suitable for imaging using conventional scintillation cameras (159 keV) and no β*^-^* radiation. However, its main limitations are its high cost and reduced availability, due to its expensive and complex production in a cyclotron. In addition, depending on the production process chosen, contaminants such as ^124^I-iodide and ^125^I-iodide may be formed increasing the dosimetry and image degradation.^4,5^

Technetium–99m, in the chemical form of pertechnetate (${\text{TcO}}_{4}^{-}$), is also used for thyroid scintigraphy and uptake.^6-17^ The similarity of volume and charge between the iodide and pertechnetate ions is the explanation for the uptake of ^99m^Tc-pertechnetate by the thyroid gland.^9,10^ ^99m^Tc-pertechnetate has been used worldwide to study the thyroid function because of a number of advantages, such as a short half-life (6 hours), short retention in the gland, and no β*^-^* radiation, thus providing low dosimetry to the thyroid gland (10,000 times less than that of ^131^I-iodide),^10^ as well as to the body as a whole.^10^ Its gamma photon of 140 keV is ideal for imaging using scintillation cameras and in addition it has low cost and is readily available.^11^

There is an international consensus that the radiopharmaceuticals of choice for thyroid gland imaging are ^99m^Tc-pertechnetate or ^123^I-iodide. Although the thyroid does not organify ^99m^Tc-pertechnetate, in the majority of cases the uptake and imaging data provide all the information needed for accurate diagnosis.^17^ In rare instances, ^123^I-iodide can subsequently be used for assessment of organification defects.

Despite these recommendations, most nuclear medicine laboratories in Brazil choose the radiopharmaceutical ^131^I-iodide to study the thyroid gland. This practice can in part be explained by the fact that there is a lack of standard values for ^99m^Tc-pertechnetate uptake by the thyroid gland. This study had the aim of standardizing a simple and fast method for performing thyroid uptake and scintigraphy and defining the ^99m^Tc-pertechnetate uptake values in normal individuals.

## METHODS

The study consisted of 47 normal individuals – 30 women and 17 men, with ages ranging from 19 to 61 years (mean of 33 years). The laboratory assessment of thyroid function was obtained via serum measurements of free thyroxine (FT_4_) or total thyroxine (T_4_) and ultra-sensitive thyrotrophin (TSH-us).

The study protocol was approved by the Ethics Committee of the School of Medical Sciences, Campinas State University.

All individuals were placed on a low iodine diet two weeks prior to the thyroid scintigraphy and uptake study. Thyroid scintigraphy and uptake were performed twenty minutes after an intravenous injection of 370 MBq (10 mCi) of ^99m^Tc-pertechnetate.

**Figure 1:**
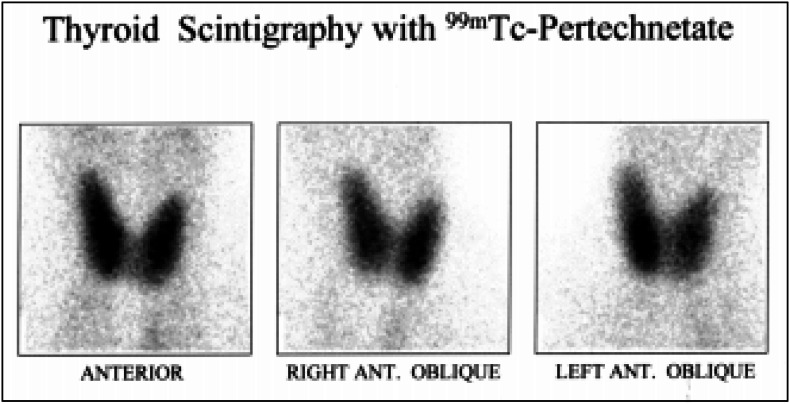
Anterior and 30-degree right and left anterior oblique images of the thyroid gland were obtained 20 minutes after an intravenous injection of 370 MBq (10 mCi) of ^99m^Tc-pertechnetate.

A scintillation camera equipped with a low-energy, high-resolution, parallel-hole collimator was used. Images were obtained on a 128 × 128 matrix and at zoom 2. Images of the syringe were obtained before and after radiopharmaceutical injection, for uptake calculation. Images of the syringe were obtained for 2 seconds and of the anterior neck for 100,000 counts. Anterior and 30° right and left anterior oblique images were obtained for studying the structure and shape of the thyroid gland (Figure 1). The time required for the acquisition of the oblique images was set at 180 seconds or 200,000 counts. A control image of the radiopharmaceutical injection site was obtained in order to check for subcutaneous infiltration that could invalidate the uptake calculation.

**Figure 2:**
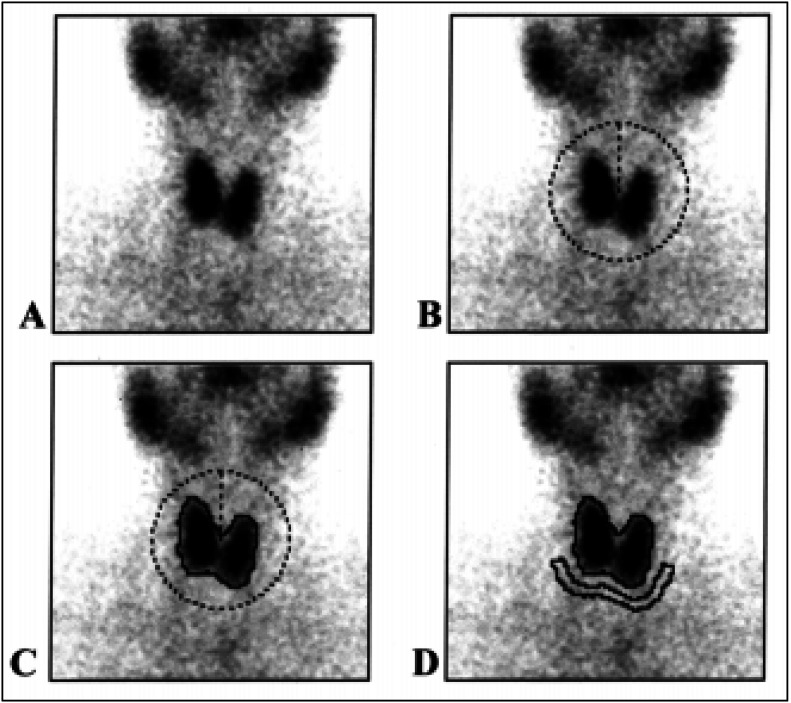
Procedure used for determining the number of counts present in the thyroid in order to obtain the ^99m^Tc-pertechnetate uptake. From an image obtained in the anterior projection (**A**), a circular region of interest (ROI) was positioned around the entire gland excluding other structures, such as the salivary glands (**B**). Using an edge detection program, the computer drew the contour of the thyroid gland (**C**) and determined the number of counts in this region. For background subtraction, another ROI was drawn immediately below the thyroid gland (**D**). Except for the positioning of the circular ROI, the entire process was automatic and took only a few seconds.

The method for the calculation of thyroid uptake, based on images of the gland and syringe counts before and after radiopharmaceutical injection, was previously described by Maisey et al.^12^ and simplified for routine use.^7^ The number of counts present in the thyroid (T) was determined by an automatic region of interest (ROI) drawn around the borders of the gland. Another ROI was drawn by the same process just below the gland for background subtraction (BG) (Figure 2). The counts in the syringe before (B) and after (A) radiopharmaceutical injection were obtained directly from the images. All counts were corrected for the acquisition time and decay of technetium-99m. The thyroid uptake (TU) was calculated according to the following equation:


$$\text{TU}=\frac{\text{T}-\text{BG}}{\text{B}-\text{A}}$$

## STATISTICAL ANALYSIS

Descriptive and exploratory analyses were made to examine the position and dispersion of the data. A frequency histogram and a box-plot were utilized for graphic presentation of the data. The Shapiro-Wilk test was utilized to evaluate the distribution of the thyroid uptake values.

## RESULTS

All the individuals were clinically euthyroid. The laboratory assessment of T_4_ (NV = 4.7 to 12 mg %) or FT_4_ (NV = 0.74 to 2.1 ng %) and TSH-us (NV = 0.38 to 6.15 mUI/ml, ultra-sensitive assay) (fluorometric enzyme immunoassay, Dade Behring Inc., Miami, USA) were within the normal limits. The thyroid uptake of ^99m^Tc-pertechnetate ranged from 0.4% to 1.7%.

The total acquisition time and the overall length of time spent by individuals in the laboratory were approximately 15 and 30 minutes, respectively.

**Figure 3:**
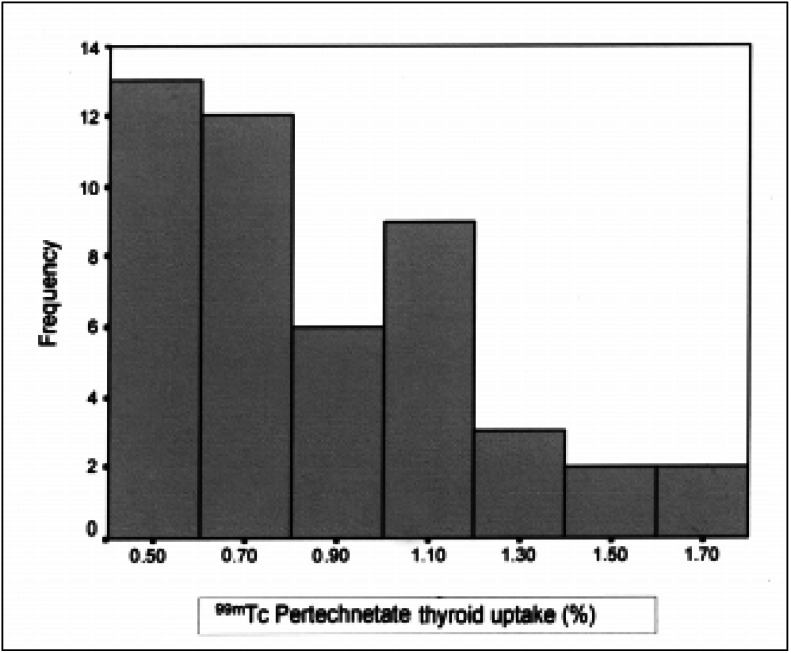
Histogram of the frequencies of measurements of thyroid uptake of the 47 normal individuals. Note the non-Gaussian distribution of the uptake values.

The frequency histogram revealed a non-Gaussian data distribution (Figure 3), confirmed by the Shapiro-Wilk test (p = 0.0002). Therefore, the mean and standard deviation (SD) values did not represent a normal distribution curve. The box-plot analysis showed that 50% of the normal individuals presented uptake values that ranged from 0.5% to 1.0%, 75% ranged from 0.4% to 1.0% and 95% ranged from 0.4% to 1.5% of the injected dose.

## DISCUSSION

The use of ^131^I-iodide for thyroid scintigraphy in nuclear medicine laboratories has been practically abolished for many years now^3^ because of the high dosimetry that the thyroid gland receives and the unsatisfactory image quality. For these reasons, the use of ^131^I-iodide for thyroid imaging in the United States has been banned by the FDA since the 1980s. Its use is restricted to extremely low doses (under 30 µCi) for some laboratories that insist on obtaining uptake values using this radiotracer or to high customized doses in capsules for differentiated thyroid carcinoma and hyperthyroidism therapy.

The radiopharmaceuticals currently chosen are ^123^I-iodide and ^99m^Tc-pertechnetate. However, ^123^I-iodide is expensive and not readily available. Therefore ^99m^Tc-pertechnetate has become the tracer of choice, since it is readily available and has a low cost. Its uptake is similar to that of iodide, although ion organification is absent. The use of ^123^I-iodide is restricted to studies that require iodide organification, as in cases of congenital dyshormonogenic hypothyroidism and organification defects in autoimmune chronic thyroiditis.^9,10^

The maximum thyroid uptake of ^99m^Tc-pertechnetate takes place 10 to 20 minutes after intravenous injection, in contrast to ^131^I-iodide, which requires a 24-hour measurement period.^13,14^

The absolute ^99m^Tc-pertechnetate uptake by the thyroid gland is low and ranges from 0.3 to 3.0%.^13,14^ It has been shown in the literature that normal values of ^99m^Tc-pertechnetate uptake depend on the technique used and on the dietary intake of iodide. Each laboratory should therefore establish its own normal values.^13,14^

The participants in this study were from the city of Campinas, in the State of São Paulo, a region where there is no iodine deficiency. The uptake values for the 47 individuals revealed a non-Gaussian distribution, as previously observed by Maisey et al.^12^ and ranged from 0.4 to 1.7%, with the lower limit being similar to that described in the literature.^12-14^ The upper limit of normality in this study was less than that previously cited in the literature^12-14^ and this difference could be due to the different techniques used and the population studied. Some of the studies that determined the range of normality were conducted by comparing cervical uptake with radioactive counts in the thigh using a thyroid probe.^15^ This instrument does not allow for precise differentiation between radiation from the thyroid gland and radiation from other structures such as the salivary glands, mouth, esophagus and blood vessels.^12^ Therefore, when a thyroid probe is positioned on the anterior region of the neck, part of the radiation detected may come from non-thyroidal structures, with marked ^99m^Tc-pertechnetate accumulation 20 minutes after injection. In this study the uptake was obtained using the image quantification technique, which delineates the thyroid tissue and excludes radiation from other structures.^16^

## CONCLUSION

The simplicity and reproducibility of this methodology and the added advantages of low dosimetry, availability and low cost make ^99m^Tc-pertechnetate the best option for thyroid scintigraphy and uptake studies.
